# Editorial: Precision Medicine in Veterinary Oncology

**DOI:** 10.3389/fvets.2021.718891

**Published:** 2021-07-14

**Authors:** Carlos Eduardo Fonseca-Alves, Chiara Palmieri, Maria Lucia Zaidan Dagli, Renée Laufer-Amorim

**Affiliations:** ^1^Institute of Health Sciences, Paulista University-UNIP, Bauru, Brazil; ^2^Department of Veterinary Surgery and Animal Reproduction, São Paulo State University–UNESP, Botucatu, Brazil; ^3^School of Veterinary Science, Gatton Campus, The University of Queensland-UQ, Brisbane, QLD, Australia; ^4^Department of Pathology, School of Veterinary Medicine and Animal Science, University of São Paulo-USP, São Paulo, Brazil; ^5^Department of Veterinary Clinic, São Paulo State University–UNESP, Botucatu, Brazil

**Keywords:** canine, feline, personalized and precision medicine, veterinary oncology, comparative medicine

Cancer is one of the most important diseases worldwide, with a global estimate of 10 million cancer-related deaths in 2020 (1). Although cancer prevention, diagnosis, and treatment have significantly improved in recent years, several cancers still represent a therapeutic challenge (2). The identification of tumor-specific characteristics has allowed for the use of monoclonal antibodies and tyrosine kinase receptor antagonist in cancer management and treatment. However, there is a growing need for models to study the efficacy of these new therapies. In this scenario, dogs and cats represent a unique opportunity for comparative oncology initiatives.

The Comparative oncology research has been strengthened in the last decade and uses animals as models to study human cancers. Initially, this concept was developed from the use of laboratory animals in cancer research and then re-shaped according to the new concept of One Health (3, 4). Since dogs develop spontaneous cancers with similarities to their human counterparts, they have been used as a model for new therapeutic interventions (5). Among the new therapeutic interventions, precision medicine has experienced unprecedented growth in recent years.

Research investigating and advancing precision medicine aims to identify individual tumor characteristics and provide treatment recommendations according to specific druggable target identification. Precision medicine covers different areas, including the identification of genomic markers, drug discovery, and health communication, supporting decision-making and providing evidence to choose the best treatment for patients (6). Although precision medicine has grown and matured tremendously in human cancer research, this concept is still new in veterinary oncology.

To fill this gap in veterinary oncology, this Research Topic is a compilation of the original and review articles that provide the most recent progress in precision oncology applied to veterinary cancer research. To instigate readers' interest in this new subject of veterinary oncology, we introduce here the main findings reported by different authors in their 10 manuscripts that form the bulk of this Research Topic.

Chibuk et al. reviewed the fundamentals of cancer genomics and provided a detailed explanation of the applications of liquid biopsies for the detection, characterization, and management of cancers in dogs. Gray et al. showed the importance of the tumor microenvironment and hypoxia as a tool for integration in a precision medicine approach. Overall, the authors reviewed several markers and technologies to be applied in precision oncology, including polymerase chain reaction assays to monitor tumor resistance and new targets for generating tyrosine kinase inhibitors. The third review paper provided a more specific direction for investigating the role of precision medicine in the diagnosis and treatment of canine mammary tumors. Valdivia et al. performed a comprehensive literature review on canine mammary tumors (CMTs), providing the most recent advances in the field of precision medicine, including the use of vasculogenic mimicry as a prognostic marker.

Interestingly, among the seven original manuscripts, three manuscripts introduced a new therapeutic perspective for canine prostate cancer. Schille et al. investigated the anti-tumor effects of PDA-66 and PDA-377 indolylmaleimides in canine prostate cancer cell lines. The authors demonstrated an interesting modification of prostate cancer cells after PDA-66 treatment and identified its anti-tumor effect through mitotic death. Another study by Kobayashi et al. investigated the antitumor effect of toceranib phosphate on two different canine prostate cancer cell lines, complemented by the cell line transcriptome after treatment. These authors identified both toceranib phosphate-sensitive and -resistant cancer cell lines. In particular, the cell line susceptible to toceranib phosphate displayed several transcriptome alterations, including dysregulation of the platelet-derived growth factor receptor pathway. Brito et al. evaluated the antitumor effect of a natural plant extract (*Synadenium grantii*) on two canine prostate cancer cell lines. These authors characterized the active principles of the plant extract using high-resolution mass spectrophotometry and demonstrated *in vitro* cytotoxicity in both prostate cancer cell lines.

Canine mammary gland tumors (CMTs) were also investigated in two original studies. Biondi et al. quantified global DNA methylation in CMTs in correlation with clinicopathological factors. Interestingly, the authors identified a specific pattern of global DNA methylation according to the tumor behavior, with hypomethylation being the most common in aggressive histological subtypes. Nakagaki et al. provided a morphological and phenotypic description of a new set of CMTs with neuroendocrine differentiation. Finally, Gambim et al. performed a meta-analysis and *in silico* analysis of dysregulated genes and proteins in canine bladder tumors, describing several genes with prognostic value in both human and canine bladder cancer.

Altogether, these manuscripts present new knowledge on the application of precision medicine in veterinary oncology that may drive a broader use of this strategy in veterinary oncology practice. The investigation of precision medicine approaches in veterinary oncology thus continues to be essential for a better strategy for cancer management and treatment in animals.

## Author Contributions

All authors listed have made a substantial, direct and intellectual contribution to the work, and approved it for publication.

## Conflict of Interest

The authors declare that the research was conducted in the absence of any commercial or financial relationships that could be construed as a potential conflict of interest.
